# Genioplasty and Hyoid Advancement by Distraction Osteogenesis for the Correction of Obstructive Sleep Apnea in a Pediatric Patient

**DOI:** 10.7759/cureus.52458

**Published:** 2024-01-17

**Authors:** Makoto Omori, Hiroatsu Iwatani, Hiroki Fukuoka

**Affiliations:** 1 Plastic Surgery, Yodogawa Christian Hospital, Osaka, JPN; 2 Department of Plastic Surgery, Kakogawa Central City Hospital, Kakogawa, JPN; 3 Department of Orthodontics, Kakogawa Central City Hospital, Kakogawa, JPN

**Keywords:** genioplasty, obstructive sleep apnea, hyoidpexy, distraction osteogenesis, genioglossus advancement

## Abstract

Advancement genioplasty is one variation of genioglossus advancement (GA) and GA is a surgical intervention that can be applied for obstructive sleep apnea (OSA) caused by hypopharyngeal collapse. The genioglossus muscle originates from the posterior surface of the midline mandible and inserts into the entire tongue mass and the body of the hyoid bone. Placing horizontal tension on the genioglossus muscle enlarges the posterior airway space. We use a modified GA that applies distraction osteogenesis to increase forward movement of the genioglossus muscle and also connects the bone transport segment to the hyoid bone with a thread to maximize the anterior movement of the hyoid bone. We used this technique on a young patient and obtained good results.

## Introduction

The algorithm proposed by Stanford University, Stanford, California, United States, is often used as a treatment guideline for obstructive sleep apnea (OSA) [1]. According to that guideline, non-surgical treatments such as continuous positive airway pressure (CPAP) and oral appliances are applied for the OSA patient first. Surgery is considered if those therapies fail or are not tolerated by the patient. Genioglossus advancement (GA) is a surgical intervention that can be applied for OSA caused by hypopharyngeal collapse. The genioglossus muscle originates from the posterior surface of the midline mandible and inserts into the entire tongue mass and the body of the hyoid bone. Placing horizontal tension on the genioglossus muscle prevents the tongue from falling back into the posterior airway space. We used a modified GA that applies distraction osteogenesis to increase forward movement of the genioglossus muscle and also connects the bone transport segment to the hyoid bone with a thread to maximize anterior movement of the hyoid bone. We used this technique on a young patient and obtained good results by increasing the efficiency of anterior traction on the genioglossus muscle. This paper reports details of the technique.

## Case presentation

The patient

**Figure 1:**
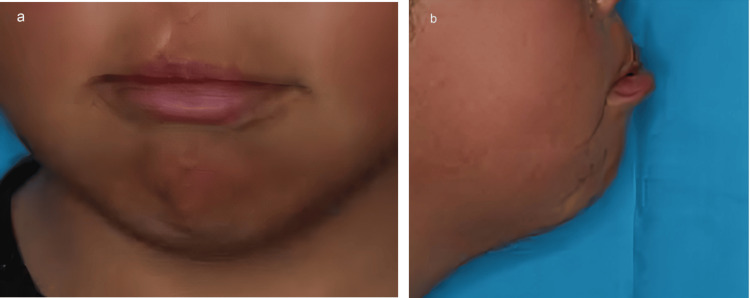
Preoperative frontal (a) and lateral (b) views.

The patient was born with a right cleft lip and palate and had undergone cheiloplasty at three months of age, palatoplasty at one year of age, and alveolar bone grafting at eight years of age (Figure 1).

**Figure 2:**
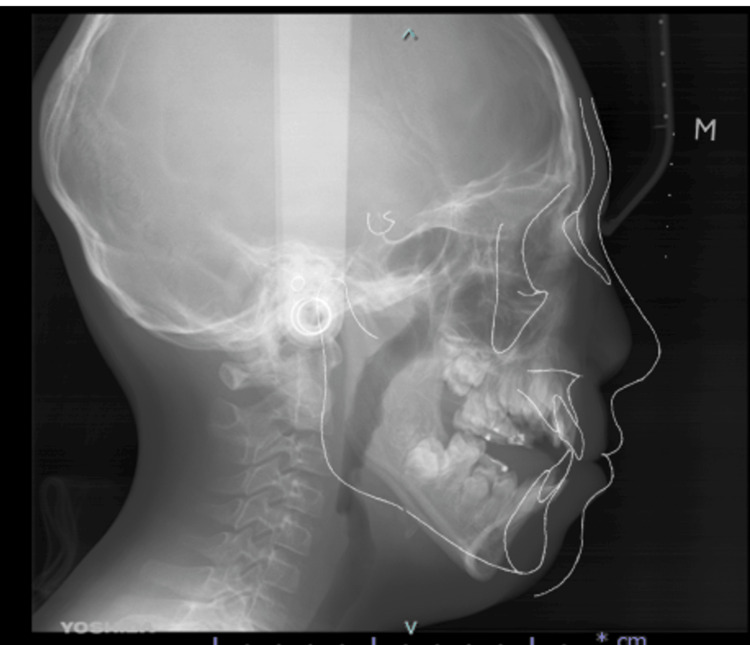
Template skeletal analysis. Template skeletal analysis shows the maxilla located posteriorly with respect to the skull base, and the mandible located further posteriorly. An open bite due to premature contact of the molar is also observed.

The patient displayed markedly enlarged tonsils, hyponasality, mouth breathing, and snoring, so tonsillectomy had also been performed at three years of age. Snoring disappeared postoperatively but started to recur around eight years of age. A tendency to wake up at night and have excessive daytime sleepiness also appeared. At nine years and two months of age, polysomnography showed an apnea-hypopnea index (AHI) of 5.4 events/hour (normal value for children: <1 event/hour), and moderate OSA was diagnosed. A sleep splint and continuous positive air pressure (CPAP) device were installed as initial treatment, but the patient did not like to use both of those appliances and stopped using them. In parallel with this treatment, a sleep MRI was performed to determine the site responsible for OSA, revealing that the airway in the nasal cavity and oropharynx was maintained and that the behind-the-tongue base was stenosed. Based on these findings, we diagnosed the tongue base as responsible for OSA. Skeletal analysis using template analysis showed that the maxilla was slightly posterior to the skull base, with the mandible further posterior. The mandible appeared rotated clockwise due to premature contact of the molars, and an open bite was observed (Figure 2).

**Figure 3:**
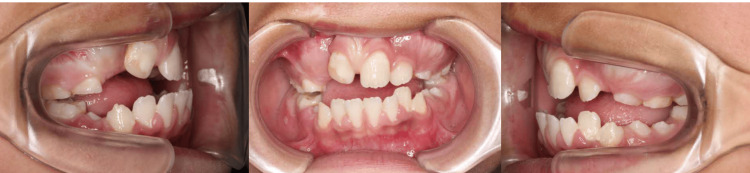
Preoperative intra-oral view. Preoperative occlusion shows the anterior open bite and Angle's class 1 occlusion at the molar.

Maxillomandibular advancement (MMA) is a powerful tool for the correction of OSA, but the patient’s AHI score was not so severe and the procedure was judged to be difficult to adapt due to the presence of unerupted tooth germs. Further, since the occlusion of the molars showed Angle's class I occlusion, we considered that the application of mandibular advancement alone would be difficult (Figure 3).

For these reasons, we decided to apply GA for this patient. To increase the efficiency of airway expansion, we connected the distraction segment and the hyoid bone with a thread to facilitate the forward movement of the hyoid bone.

Operative procedure

**Figure 4:**
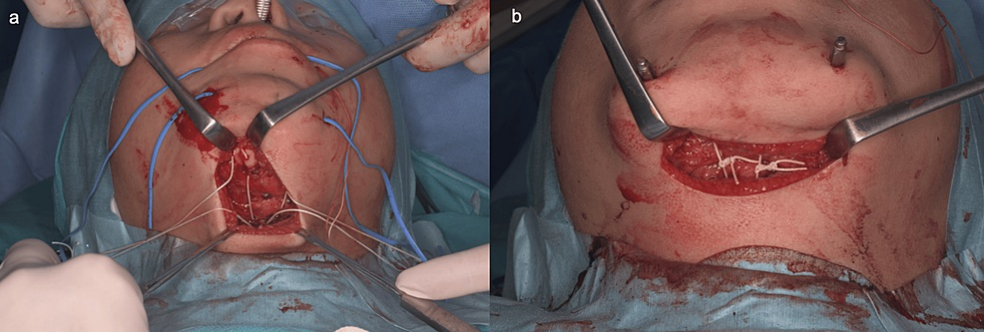
Intraoperative view. a) Gore-Tex suture* is passed between the posterior edge of the chin and the hyoid bone. b) The thread is tightened to firmly connect the chin and hyoid bone. *Gore Medical, Flagstaff, Arizona, United States

**Figure 5:**
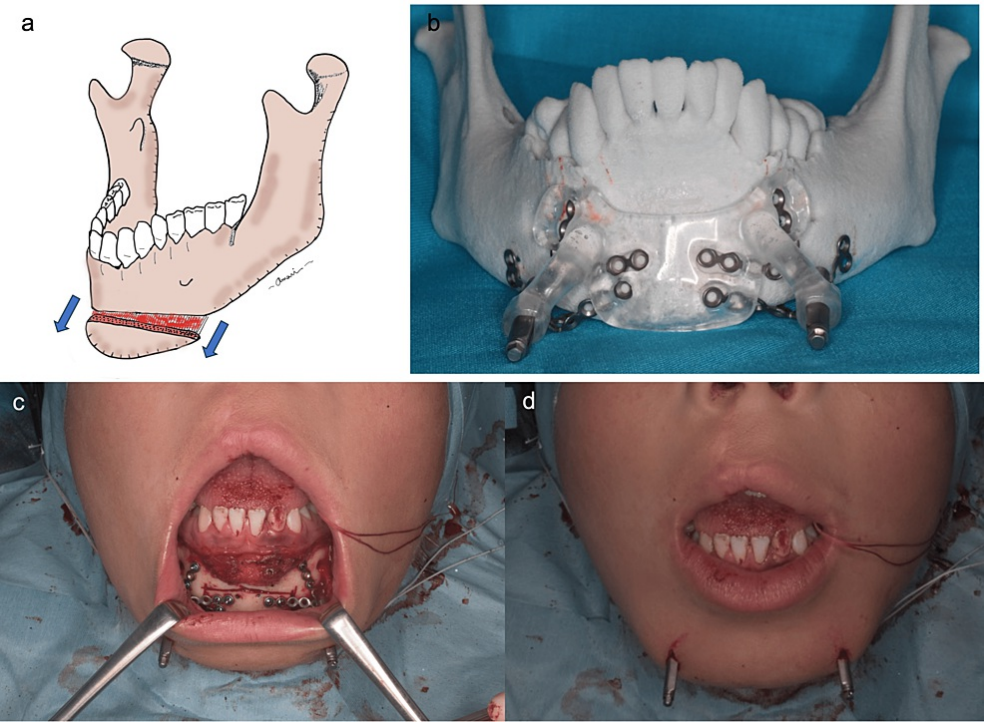
Intraoperative view. a) Illustration of the osteotomy. Image credit: Author Makoto Omori. b) Model surgery. c) The distractor is fixed to the bone. d) Immediate postoperative view.

Surgery was performed when the patient was 10 years and 0 months old. A transverse skin incision a few centimeters long was marked along the inferior border of the mandible. The skin incision was made and dissected deep to reach the hyoid bone. A CV-0 Gore-Tex thread (Gore Medical, Flagstaff, Arizona, United States) was passed through the hyoid bone. Subcutaneous dissection to reach the mandible was made anteriorly within the same skin incision. Rectangular mental osteotomy was performed and two tunnels were created by drilling a small hole in the mandibular surface at a site on what would become the mobile bone fragment, and the aforementioned Gore-Tex thread was passed through the hole to connect the hyoid bone and mandible. The mucosa between the left and right canines in the oral vestibule of the mandible was incised to reach the mental region of the mandible. The periosteum was dissected laterally up to the mental foramen on both sides and caudally to just beyond the lower border of the mandible. The osteotomy line was designed and a small incision was made in the skin through which the shaft of the distractor would pass. A Martin maxillary distractor (KLS Martin Group, Tuttlingen, Baden-Wuerttemberg, Germany) was applied to the planned position, and a screw hole for fixing the distractor was created. Next, mental osteotomy was performed using a reciprocating saw. The distractor was fixed with screws and the shaft was turned several times to confirm that the distractor could move forward without resistance. The wound was closed after ligating Gore-Tex sutures to the extent that appropriate tension was applied to the hyoid bone (Figures 4, 5).

Bone distraction

Five days postoperatively, distraction was started at a rate of 1 mm/day. The total distance of bone distraction was 12.5 mm. The distractor was removed after a two-month retention period.

Results

**Figure 6:**
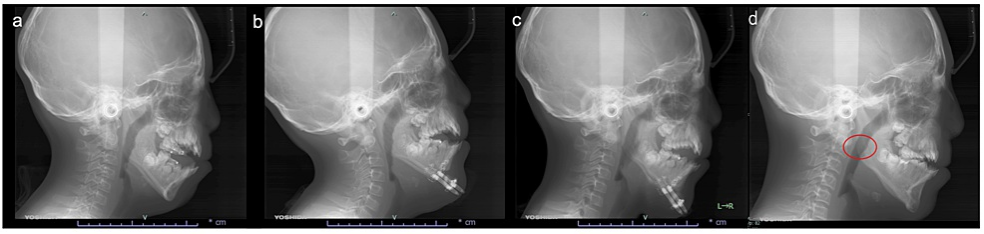
Pre- and postoperative X-rays. a) Preoperative X-ray. b) Pre-distraction X-ray. c) Post-distraction X-ray. d) Two years after surgery, showing obvious airway expansion (red circle).

Postoperative lateral cephalography showed dilatation of the airway at the hypopharynx (Figure 6).

Average saturation of percutaneous oxygen (SpO2) value and 4% oxygen desaturation index values were improved after distraction compared to when the patient had been wearing the CPAP device before surgery. Improvement was also observed in the frequency of apnea attacks (Table 1).

**Table 1:** Changes in test values before and after surgery. ODI: oxygen desaturation index

	Preoperation	Immediately after CPAP implementation	Three months postoperation
SpO_2_ (average)	96%	98%	98%
SpO_2_ (minimum)	85%	87%	92%
4% ODI	6.1	1.7	0.81
Average heart rate	73/bpm	75/bpm	83/bpm

**Figure 7:**
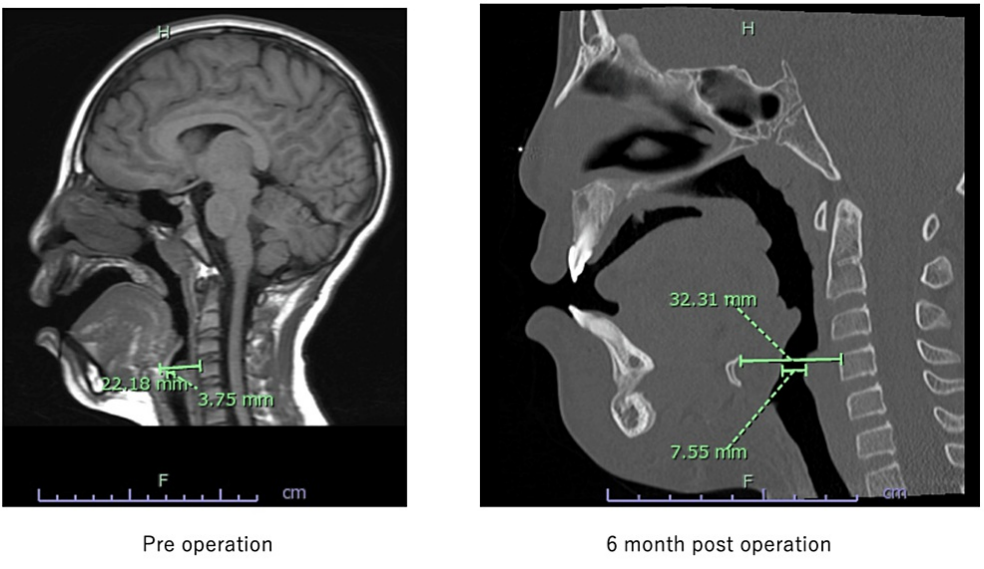
Pre- and postoperative sagittal X-ray views. Advancement of the hyoid bone and expansion of the airway are obvious.

The distance from the anterior edge of cervical vertebrae to the hyoid bone was increased from 22.18mm preoperatively to 32.31mm postoperatively. Airway space just behind the hyoid bone was increased from 3.75mm preoperatively to 7.55mm postoperatively (Figure 7). 

**Figure 8:**
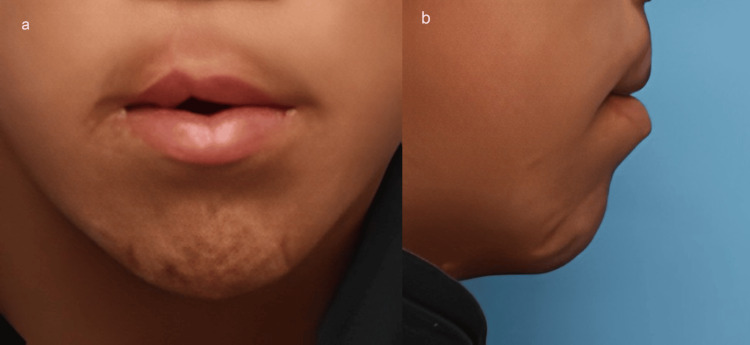
Frontal (a) and lateral (b) views 27 months postoperatively.

There was no sequela due to anterior displacement of the hyoid bone. Drug-induced sleep endoscopy showed dilatation of the pharyngeal cavity and maintenance of airway patency. Two years after the surgery, no worsening of apnea attacks had occurred (Figure 8).

## Discussion

Causative sites of OSA are classified as the nose, palate, and tongue. GA and tongue base surgery is the first-line surgery for OSA where the tongue is responsible [1]. If these treatments prove unsuccessful, MMA is indicated as a second-line surgery. In this case, we selected GA as the first-line surgery based on a comprehensive judgment of the severity of OSA, the site of origin, the results of skeletal analysis, and the age of the patient. GA is a general term for methods to expand the airway by moving the tongue body and hyoid bone forward by pulling forward the origin of the genioglossus muscle on the back of the mandible, with advancement genioplasty as one variation of this method. Limits exist to the amount of bone lengthening for one-stage advancement genioplasty, so if more movement is needed, distraction osteogenesis is indicated. Heller et al. reported mental bone lengthening by distraction osteogenesis to improve remnant OSA in a case of syndromic micrognathia after distraction osteogenesis of the mandible body [2]. The same report compared results between one-stage advancement genioplasty and distraction osteogenesis, and concluded that distraction osteogenesis is superior to one-stage advancement genioplasty.

We used a different type of distractor because of regulatory approval in Japan. The OSA improvement effect of GA is not particularly high [3], so several devices have been reported to move the hyoid bone forward and promote more effective GA [4,5]. This study used Gore-Tex threads to connect the hyoid bone with a bone transport segment, increasing the forward movement of the hyoid bone and improving the efficiency of airway expansion. This osteotomized bone transport segment does not include the genial tubercle. The reason is that cutting the bone above the location of the genial tubercle increases the possibility of damaging the tooth root and increasing the possibility of pathological fracture. Instead of not including the genial tubercle, by connecting the hyoid bone with the bone transport segment and pulling it forward, the amount of advancement of the genioglossus muscle was increased. The most effective surgical procedure for OSA caused by the tongue base is MMA. However, MMA is not usually indicated in young patients because of the risk of damage to tooth germs. The possibility remains that MMA may be required to address the future recurrence of OSA. The technique used in the present case seems unlikely to cause any inconvenience for the future performance of MMA. One drawback of the present method was that extending the chin significantly forward may lead to unfavorable changes in facial appearance, but this is easily corrected by adjusting the shape of the chin after achieving complete bone union.

## Conclusions

We performed genioplasty, connected the bone transport segment to the hyoid bone with Gore-Tex thread, and advanced the bone segment using distraction osteogenesis techniques to improve OSA. Using distraction osteogenesis techniques, significant forward movement is obtained compared to one-stage advancement genioplasty. By connecting the hyoid bone to the distraction segment, the efficacy of GA is increased.
